# TL1A promotes the postoperative cognitive dysfunction in mice through NLRP3‐mediated A1 differentiation of astrocytes

**DOI:** 10.1111/cns.14290

**Published:** 2023-06-02

**Authors:** Genghuan Wang, Jian Shen, Liping Zhai, Yingcong Lin, Qiaobing Guan, Heping Shen

**Affiliations:** ^1^ Department of Neurosurgery The Second Affiliated Hospital of Jiaxing University Zhejiang China; ^2^ Zhejiang Chinese Medical University Zhejiang China

**Keywords:** astrocytes, cognitive dysfunction, POCD, TL1A

## Abstract

**Aim:**

We investigated the mechanism, whereby tumor necrosis factor‐like ligand 1A (TL1A) mediates the A1 differentiation of astrocytes in postoperative cognitive dysfunction (POCD).

**Methods:**

The cognitive and behavioral abilities of mice were assessed by Morris water maze and open field tests, while the levels of key A1 and A2 astrocyte factors were detected by RT‐qPCR. Immunohistochemical (IHC) staining was used to examine the expression of GFAP, western blot was used to assay the levels of related proteins, and enzyme‐linked immunosorbent assay (ELISA) was used to detect the levels of inflammatory cytokines.

**Results:**

The results showed that TL1A could promote the progression of cognitive dysfunction in mice. Astrocytes differentiated into A1 phenotype, while unobvious changes were noted in astrocyte A2 biomarkers. Knockout of NLRP3 or intervention with NLRP3 inhibitor could inhibit the effect of TL1A, improving the cognitive dysfunction and suppressing the A1 differentiation.

**Conclusion:**

Our results demonstrate that TL1A plays an important role in POCD in mice, which promotes the A1 differentiation of astrocytes through NLRP3, thereby exacerbating the progression of cognitive dysfunction.

## Graphical abstract

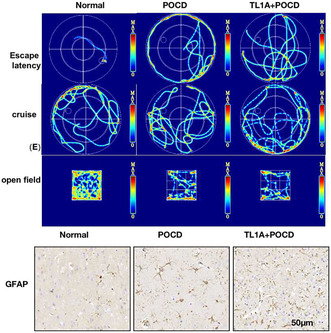

## 1 BACKGROUND

Postoperative cognitive dysfunction (POCD), as a neurological complication that occurs several days after surgery, is manifested as anxiety, insanity, as well as impairments of personality and learning and cognitive abilities.^1^, ^2^ Its mechanism is rather complicated. According to current findings, central inflammatory response can disrupt neurocytes to cause the occurrence of POCD, which resembles the case of degenerative diseases. Meanwhile, POCD also presents with oxidative stress damage, aging iron deposition, etc.^3^, ^4^ Reactive astrocytes (RAs) play an important role in regulating central nervous system diseases, which are fairly heterogeneous^5^ and can be classified into different types.^6^ In neuroinflammation, RAs can be divided into A1 and A2 phenotypes. A1 mainly promotes neuroinflammation and damages neurocytes and synapses,^7^ while A2 has the exactly opposite effect.^8^ Existing research has found that A1 appears substantially in multiple degenerative diseases, which is one major factor of cognitive dysfunction.^9^ Nevertheless, the specific mechanism of A1 differentiation in POCD remains unclear.

As a member of the tumor necrosis factor family, TL1A plays a crucial role in apoptosis and T cell activation. It has been found to act importantly in inflammatory bowel disease, neuroimmunity, and neuroinflammation.^10^ Besides, TL1A can mediate the activation of downstream signals like NLRP3 through TNFRs.^11^ NLRP3 plays a positive regulatory role in the A1 differentiation of RAs,^12^ while it remains unknown whether TL1A is associated with the RA differentiation. Hence, our study focused on the association between TL1A and RAs‐A1 in the context of POCD, which provides a new reference for the research of POCD pathomechanism.

## 2 MATERIALS AND METHODS

### 2.1 POCD model establishment and intervention

Wild‐type (WT) and NLRP3‐knockout (C57BL/6N‐Nlrp3^em1Cya^, KO) mice were purchased from Cyagen Biosciences, which were fed and watered freely in the Laboratory Animal Center of Jiaxing University. The mouse experiments complied with relevant regulations on animal ethics and welfare, and the experimental protocol was approved by the Ethics Committee.

Establishment of POCD model: A mouse model of POCD was established by splenectomy. The mice were intraperitoneally injected with 0.5 mL of normal saline and, 30 min later, with 2% pentobarbital at 25 mg/kg. Initially, the mice were weighed, anesthetized, and shaved. After placing each mouse in the lateral position and disinfecting it with iodophor, an incision was made 2–4 cm below the last left rib, and the abdominal cavity was exposed by layerwise isolation along the skin tissue. The blood vessels around the spleen were ligated, and the spleen was resected. After confirming absence of bleeding, the abdominal cavity was sutured and disinfected with iodophor. The mice were put into the cage when they woke up and fed normally. A water maze experiment was conducted on both postoperative and normal mice to determine the success of constructing a cognitive impairment model.

In Experiment 1, the WT mice were divided into three groups, namely, Normal, POCD, and POCD + TL1A (*n* = 10 per group). Mice in the Normal group underwent sham operation without splenectomy, while those in the POCD and POCD + TL1A groups underwent operation. The OCD + TL1A mice were intraperitoneally injected with recombinant TL1A cytokine (Abcam) at 5 ng/daily for 5 days prior to surgery.

In Experiment 2, the WT and KO mice were divided into WT, KO, TL1A + WT‐POCD, and TL1A + KO‐POCD groups (*n* = 10 per group). Mice in the WT and KO groups underwent sham operation without splenectomy, while those in the remaining two groups underwent operation without other drug intervention. The mice were fed and lived in the same environment.

In Experiment 3, the WT mice were divided into four groups, namely, Normal, POCD, POCD + TL1A, and POCD + TL1A + MCC950 (*n* = 10 per group). Mice in the Normal, POCD, and POCD + TL1A groups were treated in the same way as in Experiment 1, while those in the POCD + TL1A + MCC950 group were given intragastric administration of 10 mg/kg MCC950 (NLRP3 specific inhibitor) once daily in addition to intrabitoneal injection of 5 ng of TL1A since 5 days before surgery, which were administered synchronously with TL1A.

### 2.2 Morris water maze

Appropriate amount of water was added to the maze while controlling the water temperature to 25 ± 1°C. Software parameters were set. Prior to the test, the mice were placed in the laboratory instrument for 30 min so that they could have sufficient time to adapt to the experimental environment.

Positioning navigation task: The mice were put into a water maze with their heads facing a wall. If the mice climbed onto the central platform within 1.5 min, the video recording ended. If they failed to board the central platform area within 1.5 min, a wooden stick was used to guide them to swim to and board the platform, and subsequently stayed on it for 1 min. Each mouse was trained four times daily for 4 days. After the training, the mice were wiped dry and their duration of platform stay was observed.

Spatial probe task: After completing trainings in all the four quadrants, the central platform was removed, and the mice were put into the same area facing a wall. Their movement tracks within 90 s were observed, and the number of platform crossings was determined.

The Morris test was performed once weekly for 4 consecutive weeks (28 days), and the cognitive ability changes in mice were dynamically observed.

### 2.3 Open field test (OFT)

The OFT explores the behaviors and activities of mice by analyzing the distance or time of their movements in different parts of the open field, such as overall, central, and peripheral areas. The length, width, and height of the open field were 90, 90, and 15 cm, respectively. During the test, the mice were placed gently in the field center, and their movement distance and residence time in the central area within 5 min were monitored via a video tracking software. Prior to testing the next mouse, the open field was cleaned with 70% ethanol to avoid influence from the residual urine or fecal odor of the previous mouse. The test environment was kept quiet to prevent disturbance to the mice.

### 2.4 ELISA

The expression levels of inflammatory cytokines IL‐1β, IL‐6, and TNF‐α were assayed. In the animal experiment, the mouse brain tissues were isolated, cleaned twice with PBS, removed of blood vessels, membranes and other tissues, pulverized with liquid nitrogen, and then lysed on ice for 30 min with 1 mL of NP‐40 lysis buffer (Beyotime Biotechnology). The supernatant protein solutions were collected for assays. In the cell experiment, the culture medium was collected after treatment of BV2 cells, which was then centrifuged at 3000 rpm for 30 min and cryopreserved at −80°C. The cytokine levels were detected as per the protocol of the ELISA kit (Jiancheng Bioengineering Institute), and calculated by the standard curve method. The results were expressed in pg/mL.

### 2.5 RT‐qPCR

We detected the mRNA levels of A1 biomarkers (Serping1, Ggta1, C3, Psmb8, Srgn, and Amigo2) and A2 biomarkers (Clcf1, Ptx3, S100a10, Cd109, Sphk1, and Emp1) in brain tissues and RAs. Table 1 lists the primer sequences of mRNAs.

**TABLE 1:** Primer sequences for PCR.

	Forward primer (5′‐3′)	Reverse primer (5′‐3′)
Gene (A1)		
Serping1	ACAGCCCCCTCTGAATTCTT	GGATGCTCTCCAAGTTGCTC
Ggta1	GTGAACAGCATGAGGGGGTT	ACCTCGAAGACATCCCCTTT
C3	AAAAGGGGCGCAACAAGTTC	GATGCCTTCCGGGTTCTCAA
psmb8	CAGTCCTGAAGAGGCCTACG	CACTTTCACCCAACCGTCTT
Srgn	GCAAGGTTATCCTGCTCGGA	TGGGAGGGCCGATGTTATTG
Amigo2	GAGGCGACCATAATGTCGTT	GCATCCAACAGTCCGATTCT
Gene (A2)		
Clcf1	CTTCAATCCTCCTCGACTGG	TACGTCGGAGTTCAGCTGTG
Ptx3	AACAAGCTCTGTTGCCCATT	TCCCAAATGGAACATTGGAT
S100a10	CCTCTGGCTGTGGACAAAAT	CTGCTCACAAGAAGCAGTGG
Sphk1	GATGCATGAGGTGGTGAATG	GTGCTCACAAGAAGCAGTGG
Cd109	CACAGTCGGGAGCCCTAAAG	GCAGCGATTTCGATGTCCAC
Ptgs2	GCTGTACAAGCAGTGGCAAA	CCCCAAAGATAGCATCTGGA
Emp1	GAGACACTGGCCAGAAAAGC	GCAGCGATTTCGATGTCCAC
GAPDH	CCTGGAGAAACCTGCCAAGTA	TCATACCAGGAAATGAGCTTGAC

RT‐qPCR: Total RNAs were isolated from cells using TRIzol reagent (Invitrogen), which were subjected to first‐strand cDNA synthesis using a reverse transcription system (Roche) following the manufacturer's protocol. Next, PCR analysis was performed using the 7900 real‐time PCR system (Applied Biosystems, Thermo Scientific) and the SYBR Green PCR Master Mix (Roche) following the manufacturers' instructions to obtain cDNA. UC was used as a control, and the ΔΔCT method was used to present the relative RNA expression.

### 2.6 Immunohistochemical staining (IHC)

After the behavioral experiment, the mice were killed, and their brain tissues were perfused. The brain tissues were isolated, fixed in 4% paraformaldehyde, dehydrated with 15% and 30% sucrose solutions, embedded in OCT, serially sectioned at a thickness of 8 μm, and cryopreserved at −20°C. Next, the tissue sections were washed in PBS, blocked with 5% serum for 30 min, and then incubated overnight using the GFAP monoclonal antibody (1:500 TBST dilution; Abcam) at 4°C. Following three time washing in PBS, visualization was performed via the peroxidase substrate kit (Abcam), and the expression levels of GFAP were observed microscopically, We mainly observe the level of GFAP in cortical tissue.

### 2.7 Western‐Blotting

The liquid nitrogen homogenates of mouse brain tissues were used for tissue protein detection. In the cell experiment, the suspended and adherent cells were collected after treatment, which were then washed with PBS for subsequent use. The tissue homogenate cells were lysed on ice for 30 min using 1.0 mL of precooled RIPA lysis buffer (Beyotime Biotechnology), followed by protein quantification via BCA assay and adjustment of protein concentrations. After denaturation at 100°C, the protein samples were electrophoresed and transferred onto the PVDF membranes. Thereafter, the membranes were washed in TBST and incubated for 2 h with horseradish peroxidase‐conjugated secondary antibody (Abcam). Image Pro‐Plus 6.0 was used to analyze the optical density (OD), and the results were presented as OD value comparisons between the target protein and the internal GAPDH reference.

### 2.8 Cultivation of primary RAs

The primary reactive astrocytes (RAs) of mice were used for experiments. The RAs of WT and KO mice were separated. The whole brains of newborn mice aged 1–3 days were harvested, disinfected with 75% ethanol, and then the hippocampus tissues were cut into small pieces under a dissecting microscope. After digestion of the tissue pieces with 0.25% trypsin, the digestion was terminated with complete DMEM and cells were separated by filtration through a sterile 100‐μm screen. The culture medium was replaced 12–18 h later, and the cells were passaged once every 3 days, and subsequently prepared on the 1% polylysine‐coated plates.

RAs‐A1 induction conditions: stimulation with TNF‐α (30 ng/mL), IL‐1α (3 ng/mL), and C1q (400 μg/mL) for 24 h.

In Experiment 1, the RAs isolated from WT mice were used, which were divided into Control, A1, and A1 + TL1A groups. In the A1 group, A1 differentiation was performed according to the induction conditions of RAS‐A1, while in the A1 + TL1A group, 10 ng/mL TL1A was added for the cell treatment.

In Experiment 2, the RAs isolated from WT mice were divided into Control, A1, and A1 + TL1A groups, whereas the RAs isolated from KO mice were assigned to the *NLRP3* ^*−/−*^‐A1 + TL1A group. In the A1 + TL1A group, 10 ng/mL TL1A was also added for the cell treatment.

### 2.9 Statistical methods

The measurement data were expressed as (*n*, mean ± SEM). The SPSS 17.0 software was employed for data analysis and processing. After homogeneity test of variance, two independent sample t‐test was used for comparison between two groups, while one‐way ANOVA was conducted for comparison among three groups, and the subsequent pairwise comparison between groups was completed by LSD. All the above tests were two‐sided, and a difference of *p* < 0.05 signified statistical significance.

## 3 RESULTS

### 3.1 TL1A promoted the progression of POCD

**FIGURE 1:**
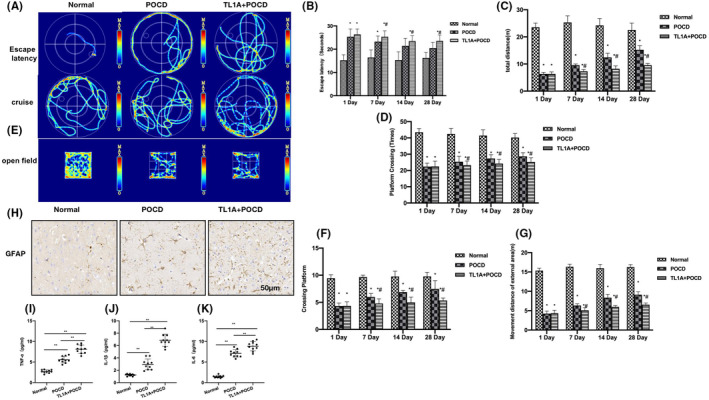
TL1A promoted the progression of POCD. (A–E) Morris water maze (*n* = 10), the Normal mice exhibited shorter escape latency, longer total movement distance and navigation time, as well as a higher number of platform crossings compared to the POCD mice. TL1A could exacerbate POCD, as manifested by the significantly longer escape latency, shorter total movement distance and navigation time, as well as less platform crossings compared to the POCD group, showing obvious differences over time. *p < 0.05 versus Normal; ^#^ *p* < 0.05 versus POCD. (F, G) OFT (*n* = 10), the movement distance was significantly longer in the Normal group than in the POCD group, while TL1A could shorten the movement distance to become shorter than that in the POCD group. *p < 0.05 versus Normal; ^#^ *p* < 0.05 versus. POCD. (H) IHC (*n* = 5), the expression of GFAP, a RAs‐A1 marker, was low in Normal, while was upregulated in POCD, leading to increased number of positive cells. TL1A could elevate the positive cell counts. (I–K) ELISA (*n* = 10), TNF‐α, IL‐6, and IL‐1β were significantly upregulated in POCD than in Normal, while TL1A could further promote the expression of inflammatory cytokines.

Morris water maze results showed that the cognitive dysfunction was insignificant in the Normal group, while was evident in the POCD group. The Normal mice exhibited shorter escape latency, longer total movement distance and navigation time, as well as higher number of platform crossings compared to the POCD mice. TL1A could exacerbate the cognitive dysfunction, as manifested by the significantly longer escape latency, shorter total movement distance and navigation time, as well as less platform crossings than the POCD group, showing obvious differences over time (Figure 1A–E). In the OFT, the mouse movement distance was significantly longer in the Normal group than in the POCD group, while it was shorter in the TL1A group than in the POCD group (Figure 1F,G). IHC staining revealed that GFAP, a RAs‐A1 marker, was lowly expressed in Normal, while was upregulated in POCD, leading to increased number of positive cells. TL1A could elevate the positive cell counts (Figure 1H). The results of inflammatory cytokine assays showed that TNF‐α, IL‐6, and IL‐1β were significantly upregulated in POCD than in Normal, and TL1A could further facilitate the expression of inflammatory cytokines (Figure 1K).

### 3.2 Effects of TL1A on the RAs‐A1 differentiation

**FIGURE 2:**
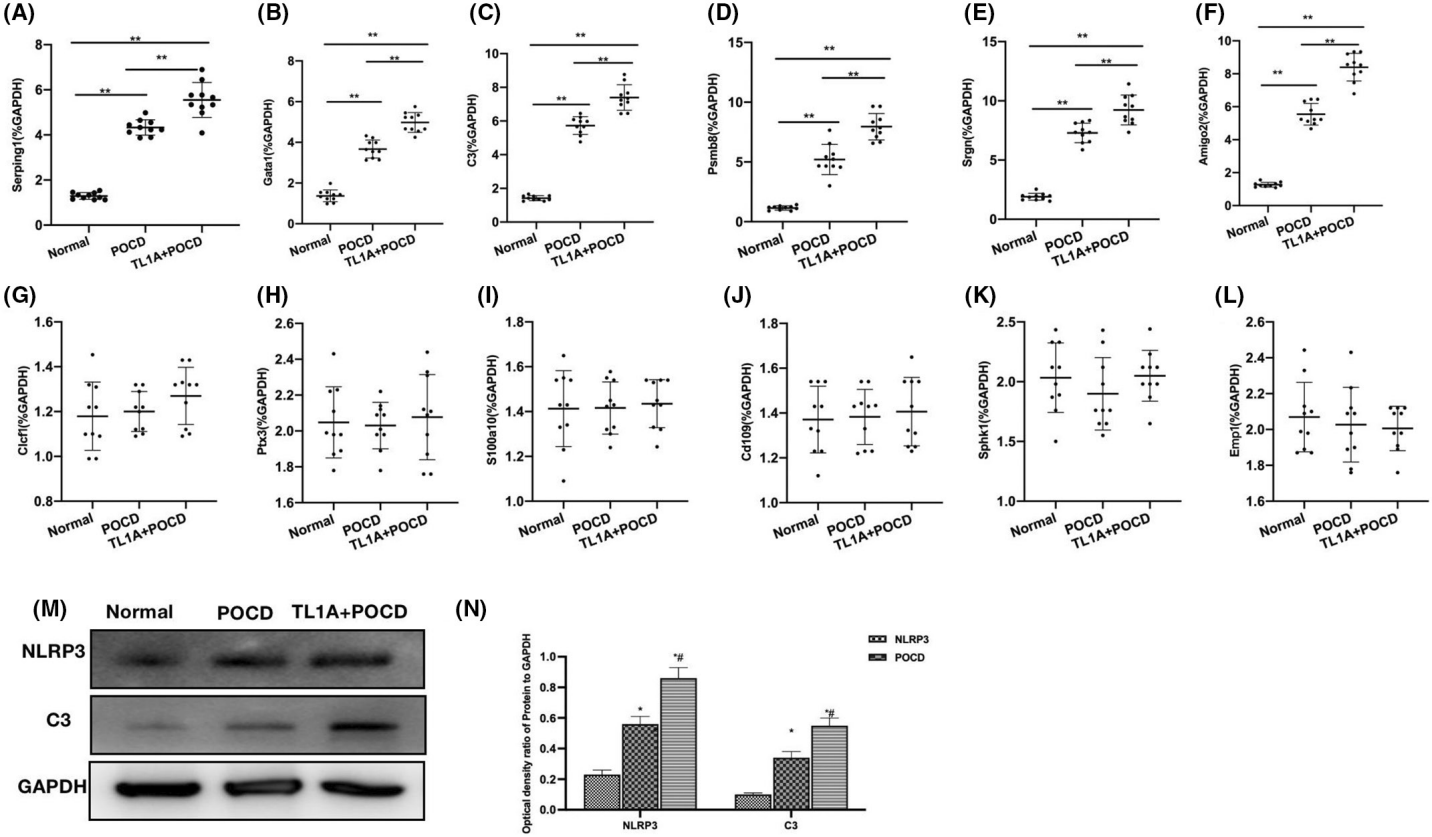
Effects of TL1A on the RAs‐A1 differentiation. (A–F) A1 RASmRNA (*n* = 10), the mRNA levels of A1 biomarkers in POCD were significantly higher than those in Normal. TL1A could further upregulate these mRNA levels. **p < 0.0 inter‐group comparisons. (G–L) A2 RASmRNA (*n* = 10), the mRNA levels of A2 biomarkers did not change significantly, showing absence of inter‐group differences. (M, N) Relative protein expressions (*n* = 3), the POCD group exhibited higher levels of NLRP3, C3 compared to the Normal group. TL1A could further elevate the levels of NLRP3, C3. *p < 0.05 versus Normal; ^#^ *p* < 0.05 versus POCD.

The mRNA levels of A1 biomarkers (Serping1, Ggta1, C3, Psmb8, Srgn, and Amigo2) and A2 biomarkers (Clcf1, Ptx3, S100a10, Cd109, Sphk1, and Emp1) were detected. The results showed significantly upregulated mRNA levels of A1 biomarkers in POCD, which were higher than those in Normal. TL1A could further upregulate these mRNA levels (Figure 2A–F). Contrastively, insignificant changes were noted in the mRNA levels of A2 biomarkers, showing absence of inter‐group differences (Figure 2G–L). Protein assays revealed that the levels of NLRP3, C3, in POCD were higher than in Normal, TL1A could further upregulate the levels of NLRP3, C3, (Figure 2M,N).

### 3.3 NLRP3 knockout could antagonize the action of TL1A and ameliorate the POCD in mice

**FIGURE 3:**
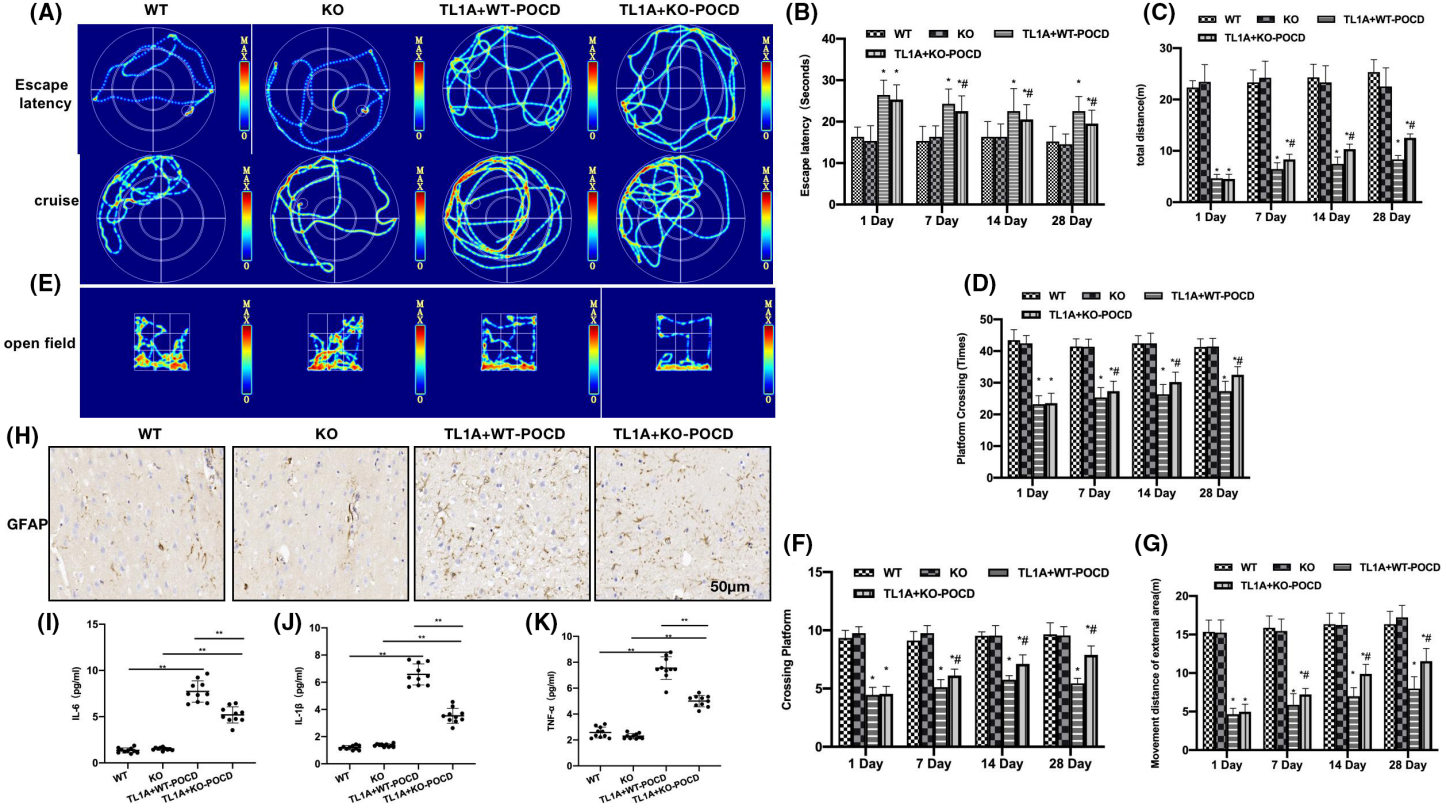
NLRP3 knockout could antagonize the action of TL1A and ameliorate the POCD in mice. (A–E) Morris (*n* = 10), obvious cognitive dysfunction appeared in TL1A + WT‐POCD and TL1A + KO‐POCD mice. Compared to the TL1A + WT‐POCD group, the TL1A + KO‐POCD group exhibited shorter escape latency, longer total movement distance and navigation time, as well as higher number of platform crossings. *p < 0.05 versus WT; ^#^ *p* < 0.05 versus TL1A + WT‐POCD. (F, G) OFT (*n* = 10), the total movement distance was longer in the TL1A + KO‐POCD group than in the TL1A + WT‐POCD group. *p < 0.05 versus WT; ^#^ *p* < 0.05 versus TL1A + WT‐POCD. (H) IHC (*n* = 5), no distinctly positive cells were found in WT and KO mice, while the number of positive cells in TL1A + KO‐POCD was significantly lower than that in TL1A + WT‐POCD. (I–K) ELISA (*n* = 10), the levels of inflammatory cytokines in TL1A + KO‐POCD were significantly lower than those in TL1A + WT‐POCD. **p < 0.0 inter‐group comparisons.

The Morris water maze test revealed absence of obvious cognitive dysfunction in WT and KO mice, proving that the NLRP3 knockout did not affect POCD significantly. Obvious cognitive dysfunction was noted in the TL1A + WT‐POCD and TL1A + KO‐POCD mice, with the TL1A + KO‐POCD mice being less dysfunctional than the TL1A + WT‐POCD mice, as manifested by shortened escape latency, prolonged total movement distance and navigation time, as well as increased number of platform crossings (Figure 3A–E). OFT results showed that the total movement distance was longer in the TL1A + KO‐POCD group than in the TL1A + WT‐POCD group (Figure 3F,G). GFAP assay revealed absence of distinctly positive cells in WT and KO, while the number of positive cells in TL1A + KO‐POCD was significantly lower than that in TL1A + WT‐POCD (Figure 3H). According to the results of ELISA assays, the levels of inflammatory cytokines in the TL1A + KO‐POCD group were significantly reduced compared to the TL1A + WT‐POCD group (Figure 3I–K).

### 3.4 Effects of NLRP3 knockout on the TL1A‐induced RAs‐A1 differentiation

**FIGURE 4:**
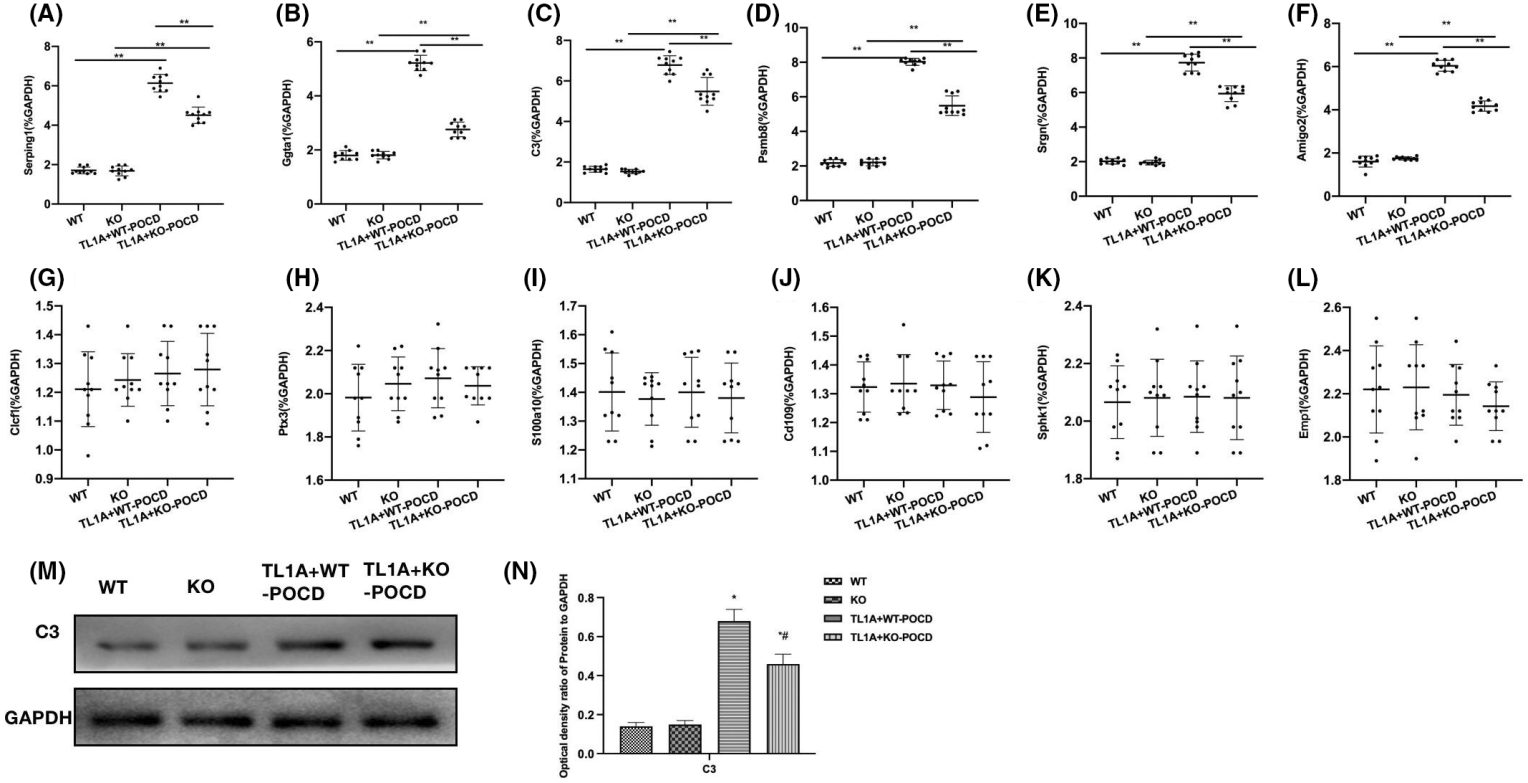
Effects of NLRP3 knockout on the TL1A‐induced RAs‐A1 differentiation. (A–F) A1 RA mRNA (*n* = 10), the mRNA levels of Serping1, Ggta1, C3, Psmb8, Srgn, and Amigo2 were lower in the TL1A + KO‐POCD group than in the TL1A + WT‐POCD group. **p < 0.0 inter‐group comparisons. (G–L) A2 RA mRNA (n = 10), the mRNA levels of A2 RA biomarkers changed insignificantly, showing absence of inter‐group differences. (M, N) Relative protein expressions (*n* = 3), compared to the TL1A + WT‐POCD group, the expressions of C3 were significantly lowered in the TL1A + KO‐POCD group. *p < 0.05 versus WT; ^#^ *p* < 0.05 versus TL1A + WT‐POCD.

The assay results of A1 RA biomarkers showed that the NLRP3 knockout could lower the mRNA levels. Compared to the TL1A + WT‐POCD group, the mRNA levels of Serping1, Ggta1, C3, Psmb8, Srgn, and Amigo2 declined in the TL1A + KO‐POCD group (Figure 4A–F). The A2 RA mRNA detection revealed insignificant differences between groups (Figure 4G–L). Protein assay results showed that compared to the TL1A + WT‐POCD group, the expressions of C3 were significantly reduced in the TL1A + KO‐POCD group (Figure 4M,N).

### 3.5 MCC950 could inhibit the action of TL1A and reduce the RAs‐A1 differentiation

**FIGURE 5:**
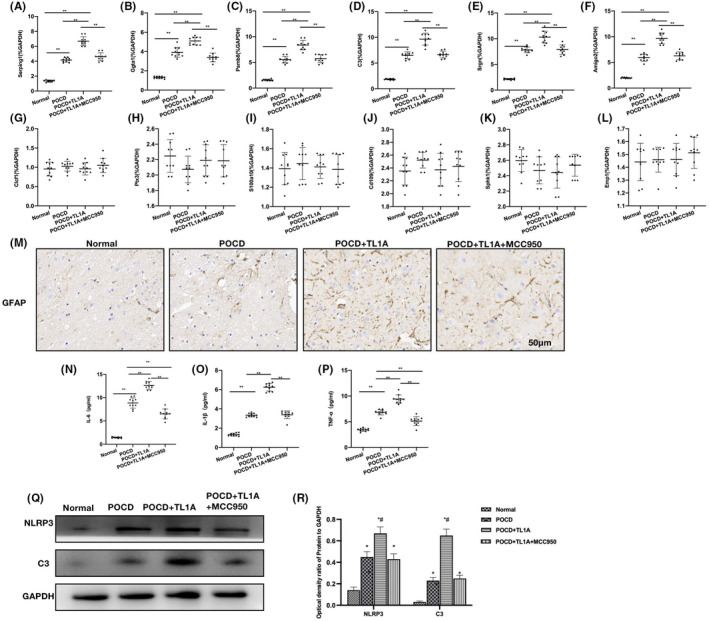
MCC950 could inhibit the action of TL1A and reduce the RAs‐A1 differentiation. (A–F) A1 RA mRNA (*n* = 10), MCC950 could inhibit the RAs‐A1 differentiation, as manifested by significantly declined mRNA levels of A1 RA biomarkers in the POCD‐TL1A + MCC950 group than in the POCD‐TL1A group. **p < 0.0 inter‐group comparisons. (G–L) A2 RA mRNA (n = 10), the mRNA levels of A2 RA biomarkers changed insignificantly, showing absence of inter‐group differences. (M) IHC (*n* = 5), GFAP assay revealed significantly lower number of positive cells in the POCD‐TL1A + MCC950 group than in the POCD‐TL1A group. (N–P) ELISA (*n* = 10), ELISA assay also revealed significantly declined levels of inflammatory cytokines in the POCD‐TL1A + MCC950 group than in the POCD‐TL1A group. **p < 0.0 inter‐group comparisons. (Q, R) Relative protein expressions (*n* = 3), MCC950 could lower the levels of NLRP3, C3, showing significant differences from the POCD‐TL1A group. *p < 0.05 versus Normal; ^#^ *p* < 0.05 versus POCD.

We used MCC950, an NLRP3 inhibitor, to block the action of NLRP3, and the results showed that MCC950 could antagonize the action of TL1A and inhibit the RAs‐A1 differentiation. Compared to the POCD‐TL1A group, the A1 RA mRNA levels in the POCD‐TL1A + MCC950 group decreased significantly (Figure 5A–F), while the A2 RA mRNA levels showed insignificant changes (Figure 5G–L). In GFAP assay, the number of positive cells in POCD‐TL1A + MCC950 was significantly lower than that in POCD‐TL1A (Figure 5M). Inflammatory cytokine assays also revealed significantly declined cytokine levels in the POCD‐TL1A + MCC950 group than in the POCD‐TL1A group (Figure 5N–P). According to the protein assay results, MCC950 could reduce the levels of NLRP3, C3, showing significant differences from the POCD‐TL1A group (Figure 5Q,R).

### 3.6 TL1A promoted the primary RAs‐A1 differentiation in vitro

**FIGURE 6:**
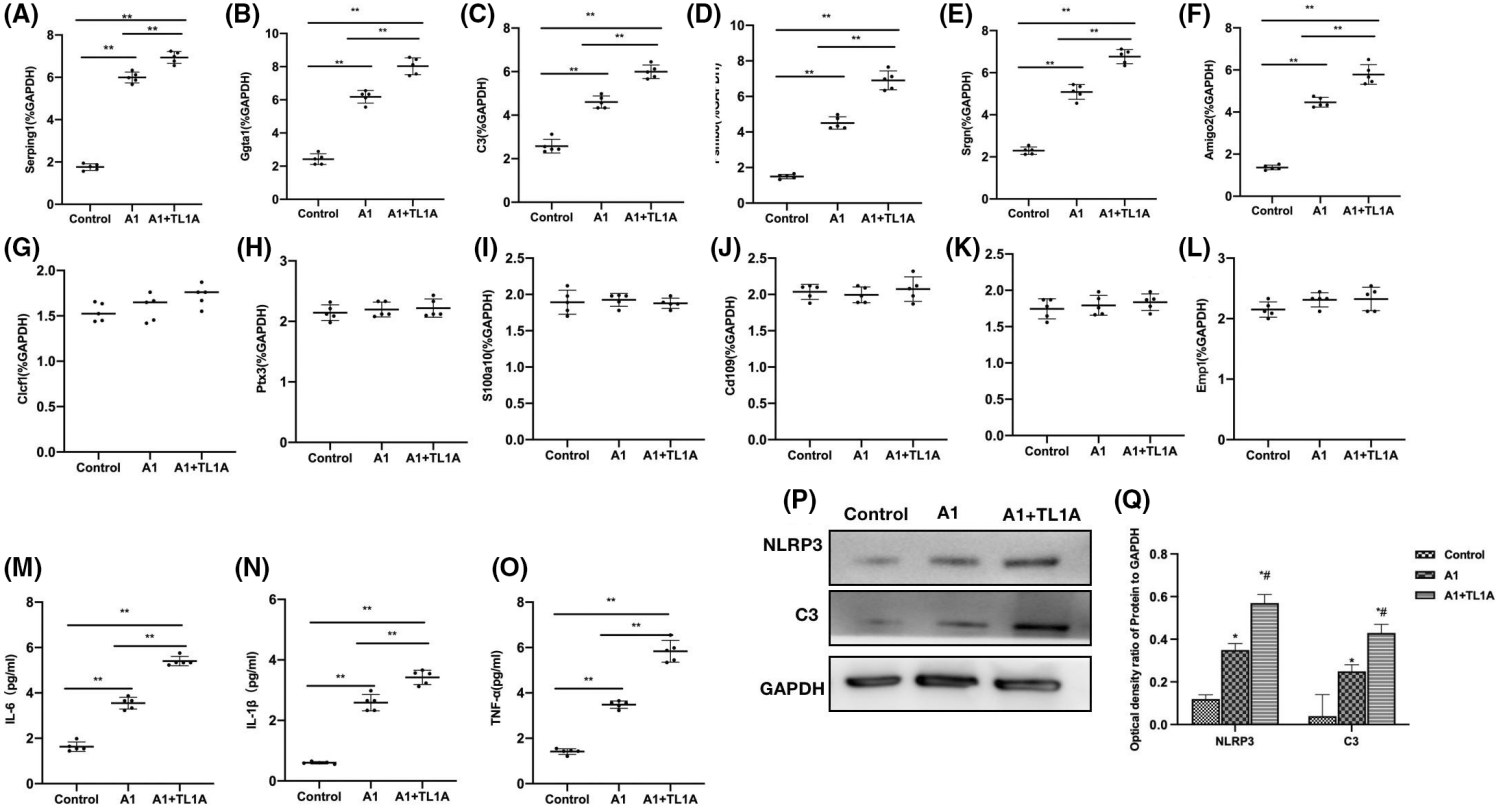
TL1A promoted the primary RAs‐A1 differentiation in vitro. (A–F) A1 RA mRNA (*n* = 5), TL1A could promote the RAs‐A1 differentiation and elevate the mRNA levels of A1 RA biomarkers. **p < 0.0 inter‐group comparisons. (G–L) A2 RA mRNA (*n* = 5), the mRNA levels of A2 RA biomarkers changed insignificantly, showing the absence of inter‐group differences. (M–O) ELISA (*n* = 5), TL1A could promote the secretion of inflammatory cytokines by RAs, as manifested by significantly upregulated cytokine levels in the A1 + TL1A group than in the A1 group. **p < 0.0 inter‐group comparisons. (P, Q) Relative protein expressions (*n* = 3), TL1A could promote the expressions of NlRP3, C3. *p < 0.05 versus Control; ^#^ *p* < 0.05 versus A1.

Reactive astrocytes (RAs) were successfully differentiated into the A1 phenotype so that the A1 RA mRNA levels were significantly upregulated, which were higher than those in the Control group. TL1A could promote the RAs‐A1 differentiation and elevate the mRNA levels (Figure 6A–F). Contrastingly, the A2 RA mRNA levels showed insignificant changes (Figure 6G–L). Inflammatory cytokine assays revealed that TL1A could promote the cytokine secretion by RAs. Compared to the A1 group, the cytokine levels in the A1 + TL1A group were significantly upregulated (Figure 6M–O). Protein assays also demonstrated that TL1A could promote the expressions of NlRP3, C3 (Figure 6P,Q).

### 3.7 NLRP3 knockout could inhibit the action of TL1A in vitro

**FIGURE 7:**
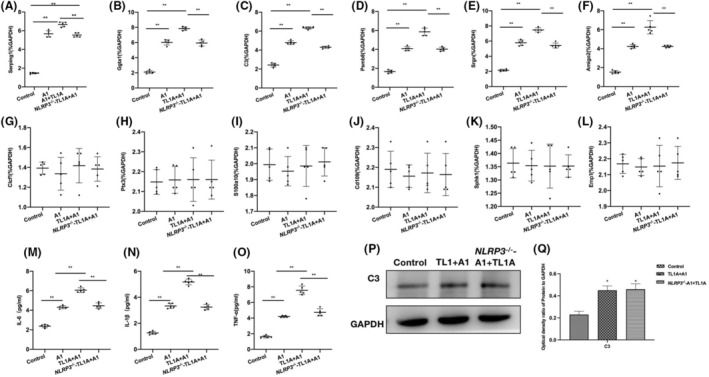
NLRP3 knockout could inhibit the action of TL1A in vitro. (A–F) A1 RAs mRNA (*n* = 5), the A1 differentiation was inhibited in the *NLRP3* ^*−/−*^‐A1 + TL1A group, where the mRNA levels of A1 biomarkers were significantly lower than those in the A1 + TL1A group. **p < 0.0 inter‐group comparisons. (G–L) A2 RAs mRNA (*n* = 5), the mRNA levels of A2 biomarkers changed insignificantly, showing absence of inter‐group differences. (M–O) ELISA (*n* = 5), the inflammatory cytokine levels were significantly lower in *NLRP3* ^*−/−*^‐A1 + TL1A than in A1 + TL1A. **p < 0.0 inter‐group comparisons. (P, Q) Relative protein expressions (*n* = 3), the inter‐group differences in C3 were insignificant after knockout of NLRP3. *p < 0.05 versus Control; ^#^ *p* < 0.05 versus A1.

Comparison of RAs between WT and KO mice found that the A1 differentiation was inhibited in the *NLRP3* ^*−/−*^‐A1 + TL1A group, leading to significantly lower mRNA levels of A1 biomarkers than those in the A1 + TL1A group (Figure 7A–F). Meanwhile, no significant changes were noted in the A2 mRNA levels (Figure 7G–L). Inflammatory cytokine assays revealed that the cytokine levels were significantly lower in the *NLRP3* ^*−/−*^‐A1 + TL1A group than in the A1 + TL1A group (Figure 7M–O). According to the protein assay results, the inter‐group differences in C3 were insignificant after knockout of NLRP3 (Figure 7P,Q).

## 4 DISCUSSION

Postoperative cognitive dysfunction (POCD) is a common complication following anesthesia, especially among elderly patients aged 65+ years and those who have undergone major surgery, with high morbidity and certain mortality.^13^ The high incidence of POCD in elderly patients is due to the interaction of multiple factors such as aging of the nervous system and poor recovery from trauma and anesthesia.^14^, ^15^ POCD is a disease with complex etiology. Although POCD has been researched for decades, with accumulation of substantial animal models and clinical studies, its pathogenesis and pathophysiology remain unclear, which may be associated with inflammation caused by postoperative trauma.^16^, ^17^ Recently, reactive RAs have been reported to be classified into types A1 and A2 similar to the microglia cells.^18^ RAs‐A1 upregulate a variety of synaptic destructive trophic factors to elevate the expressions of inflammatory cytokines, while RAs‐A2 upregulates neurotrophic factors to promote the neuronal and synaptic repair.^19^, ^20^ Research of depression has demonstrated that NLRP3 is involved in the A1 differentiation of RAs,^21^ and such effect is associated with the activation of downstream NF‐κB signaling. However, related work has never been reported in POCD.

Studies have found that TL1A, a member of tumor necrosis family, is involved in the regulation of tumor, inflammation, and immunity, which exerts its role primarily via the DR3 ligand,^22^, ^23^ and is capable of binding to TNFRs to promote the activation of downstream signals. NLRP3, a major response protein in signaling downstream of TNFRs, can mediate the inflammatory effect via NF‐κB, which can also promote the maturation of inflammatory cytokines via Caspase1. According to previous findings, NLRP3 can even promote the A1 differentiation of RAs to induce inflammation. To explore the role of TL1A in POCD, we injected TL1A into POCD mice, and the results showed that TL1A could promote the progression of POCD in mice. Morris and OFT are the common methods for examining the cognitive and behavioral abilities of mice. With the extension of time, the cognitive dysfunction in TL1A mice was aggravated. However, we believe that there is inherent trauma in mice after splenectomy, which can affect the results of the Morris escape latency experiment and prolong the escape time. These results need to be considered. However, after comparing TL1A, we have come to this conclusion. Meanwhile, the number of GFAP positive cells in the cerebral cortex was upregulated, so was the expressions of inflammatory cytokines, indicating that TL1A promoted the activation of RAs and the tissue inflammatory response. Serping1, Ggta1, C3, Psmb8, Srgn, and Amigo2 were highly expressed in RAs‐A1. RT‐qPCR revealed significant upregulation of A1 biomarkers, proving that TL1A promoted the differentiation of RAs to A1. The A2 biomarkers, on the other hand, did not change significantly, indicating that TL1A had insignificant effect on RAs‐ A2. Protein assay results showed that TL1A facilitated the activation of NLRP3 which was linked to the A1 differentiation of RAs. When we used NLPR3‐knockout mice and NLRP3 inhibitor, the effects of TL1A were significantly inhibited, causing amelioration of murine cognitive dysfunction and suppression of A1 differentiation. These results proved that NLRP3 is the downstream signal of TL1A, and that the TL1A‐mediated RASA1 differentiation requires the involvement of NLRP3. To clarify the association between RAs and TL1A, we conducted in vitro experiments using primary RAs. The RA experiments also proved that TL1A could promote the A1 differentiation and NLRP3 activation in vitro. After the use of NLRP3‐knockout RAs, the effects of TL1A were inhibited, so were the expressions of inflammatory cytokines. The in vivo and in vitro results were consistent.

## 5 CONCLUSION

We find that TL1A can promote the A1 differentiation of RAs via NLRP3 while aggravating the POCD. RAs‐A1 is a major pathological factor in the progression of POCD, while TL1A is an important cytokine in such progression. We provide a new reference for the pathological study of POCD.

## AUTHOR CONTRIBUTIONS

Genghuan Wang and Yingcong Lin are mainly responsible for the operation of the experiment, the acquisition of relevant data, and the statistical distraction of the data; JianShen, Liping Zhai are mainly responsible for literature review, project coordination, partial data analysis, and article writing; Genghuan Wang and Qiaobing Guan are mainly responsible for project development, financial support, overall project proposal, and experimental design.

## CONSENT FOR PUBLICATION

All authors approval published the article.

## Data Availability

Data sharing not applicable to this article as no datasets were generated or analysed during the current study.
